# Genetics and Pathogenicity of Influenza A (H4N6) Virus Isolated from Wild Birds in Jiangsu Province, China, 2023

**DOI:** 10.1155/2024/7421277

**Published:** 2024-02-14

**Authors:** Xingdong Song, Jingman Tian, Minghui Li, Xiaoli Bai, Zhiguo Zhao, Jianzhong Shi, Xianying Zeng, Guobin Tian, Yuntao Guan, Pengfei Cui, Guohua Deng, Liling Liu, Hongliang Chai, Yanbing Li, Hualan Chen

**Affiliations:** ^1^College of Wildlife and Protected Area, Northeast Forestry University, Harbin 150040, Heilongjiang Province, China; ^2^State Key Laboratory for Animal Disease Control and Prevention, Harbin Veterinary Research Institute, Chinese Academy of Agricultural Sciences, Harbin 150069, Heilongjiang Province, China

## Abstract

During the routine surveillance, we isolated nine H4N6 subtype avian influenza viruses (AIVs) in Jiangsu Province, China, in March 2023. Phylogenetic analysis revealed that nine H4N6 viruses belonged to the Eurasian lineage and underwent complex genetic recombination among Asian countries during their evolution. It is particularly noteworthy that the PB2 and PB1 genes of our representative virus were descended from clade 2.3.4.4b H5 high-pathogenic AIVs in Japan. Mutations of D3V and D622G in PB1, N66S in PB1-F2, N30D, I43M, and T215A in M1, and P42S and I106M in NS1 were observed in nine isolates, which may increase the pathogenicity of the viruses in mice. The receptor binding analysis showed that the tested H4N6 virus could bind to both avian-type and human-type receptors. Vitro infection kinetics revealed that the representative virus could efficiently replicate in mammalian cells, including MDCK and 293T cells. Pathogenicity tests in mice indicated that the representative virus could replicate in nasal turbinates and lungs without prior adaptation. Our data reveal the potential public health issues represented by H4N6 viruses from wild birds and highlight the need to strengthen routine surveillance of wild birds.

## 1. Introduction

Avian influenza virus (AIV) is an enveloped, single-stranded, negative-sense RNA virus belonging to influenza A genus of the Orthomyxoviridae family [1], which is classified into subtypes based on antigenic properties of their surface glycoproteins hemagglutinin (HA) and neuraminidase (NA) [2], with 16 HA subtypes (H1–H16) and nine NA subtypes (N1–N9) having been identified in wild aquatic birds so far [1, 3]. According to molecular signatures within their HA segment and as a result of clinical disease in chickens, AIVs can also be divided into low-pathogenicity avian influenza virus (LPAIV) and high-pathogenicity avian influenza virus (HPAIV). Although the pathogenicity of LPAIV to poultry is not as severe as that of HPAIV, it cannot be ignored due to its wide distribution and complex biological characteristics. Moreover, there have also been cases of LPAIV directly infecting humans across the interspecies barrier. As early as 1998, the first human case with H9N2 AIV infection was reported in Hong Kong, China [4]. In 2010, H10N7 virus was found to cause human infection in Australia [5]. Subsequently, the first case of human infection with H10N8 AIV was confirmed in China in 2013 [6]. Recently, the H10N3 virus was also reported to infect humans in China in 2021 [7]. In February 2013, the first human case of H7N9 LPAIV infection was identified in Shanghai [8], and the Taiwan region reported the first case of human infection with the H6N1 virus in June of the same year [9]. In early 2018, a 68-year-old woman was confirmed to be infected with the H7N4 virus [10], and a novel reassortant H3N8 AIV was identified in a 4-year-old boy in China last year [11].

Although the H4 subtype AIV is classified as an LPAIV, it spreads widely in wild bird species worldwide, has been found in poultry [12–14], and can even infect mammals such as pigs [15–17]. Some studies have shown that the H4 AIVs can infect mice without any prior adaptation [12] and were efficiently transmitted among guinea pigs by direct contact [13]. In addition, H4 AIVs can bind to avian-like receptors (*α*−2,3-sialic acid receptors) and even acquire the ability to bind to human-like receptors (*α*−2,6-sialic acid receptors) [18]. Therefore, we should pay great attention to the public health concerns from the H4 viruses.

In this study, we isolated nine H4N6 subtype AIVs from mallards in Yancheng City, Jiangsu Province, China in March 2023, and we analyzed the phylogenetic evolution characteristics, receptor binding preference, replication capacity in vitro, and pathogenicity in mice of the H4N6 virus. Our data revealed that the H4N6 viruses posed a potential threat to public health security, and it is necessary to strengthen regular surveillance of the H4N6 viruses circulating in wild birds.

## 2. Materials and Methods

### 2.1. Ethics Statements and Biosecurity

All experimental animal procedures were strictly executed in adherence to recommendations in the Guide for the Care and Use of Laboratory Animals of the Ministry of Science and Technology of the People's Republic of China. The processing of all swab samples collected during the surveillance, as well as studies on live H4 viruses, were carried out in the enhanced biosafety level 2 (BSL2+) laboratory approved for use by the Harbin Veterinary Research Institute (HVRI) of the Chinese Academy of Agricultural Sciences (CAAS). The study protocol was authorized by HVRI, CAAS.

### 2.2. Sample Collection

During the routine monitoring of wild birds in 2023, we collected 1,000 fresh fecal samples from wild bird habitats in China. These samples were put in a phosphate-buffered solution (PBS) (pH 7.0) containing penicillin, streptomycin, and 10% glycerin and were stored at a low temperature for transport.

### 2.3. Virus Isolation and Identification

After the samples were oscillated and centrifuged, they were inoculated into 10-day-old chicken embryos. Following incubation at 37°C for 72 hr, the allantoic fluid was harvested for the hemagglutination test by using 1% chicken red blood cells. The HA subtypes of the positive allantoic fluid were confirmed by the hemagglutinin inhibition (HI) test using H1–H16 monofactor serum, and NA subtypes were directly determined using RT-PCR with N1–N9-specific primers [19].

Mitochondrial DNA was extracted from the samples using the QIAamp Fast DNA Stool Mini Kit (Qiagen, Hilden, Germany). Then, the obtained DNA served as templates for PCR reactions with specific primers. PCR products were sequenced for species identification using BLAST (http://blast.ncbi.nlm.nih.gov/Blast.cgi).

### 2.4. Genome Sequencing

Viral RNA was extracted from the positive allantoic fluid using a QIAamp viral RNA mini kit (Qiagen, Hilden, Germany) and then reverse transcribed into cDNA with specific primers. The obtained cDNA served as the template for RT-PCR to amplify each gene fragment of the influenza A virus. The amplified DNA fragments were purified and then sequenced on the Applied Biosystems DNA analyzer at the HVRI. The sequence assembly was performed using Seqman software in the DNASTAR Lasergene 7.1 package. The primer sequences used above are available upon request.

### 2.5. Phylogenetic Evolution Analysis

To better understand the phylogenetic evolution of H4N6 subtype AIVs from wild birds, Bayesian phylogenetic analysis for eight segments of H4N6 isolates was performed by Markov chain Monte Carlo (MCMC) method using BEAST v1.10.4 [20]. Reference sequences were obtained from the NCBI Influenza Virus database (http://www.ncbi.nlm.nih.gov/genomes/FLU/FLU.html) and GISAID EpiFlu database (http://platform.gisaid.org), and the coding regions of these sequences were aligned using MAFFT v7.490 [21]. Before the analysis, the temporal signal of our dataset was assessed by root-to-tip regression using TempEst v1.5.3 [22]. An uncorrelated lognormal relaxed molecular clock and an SRD06 nucleotide substitution model were implemented using the MCMC method run for 200 million generations and sampled every 10,000 generations. Runs were assessed in Tracer v1.7.2 [23] for sufficient convergence (effective sample size >200), and a maximum clade credibility (MCC) tree was generated in TreeAnnotator v1.10.4 after removing the first 10% of runs as burn-in. The obtained MCC tree was edited by FigTree v1.4.4.

The maximum likelihood (ML) trees of eight gene segments of our isolates were constructed using MEGA v7.0 with 1,000 bootstraps. The sequence homology cutoff of >97% was used to categorize the group of each gene segment.

### 2.6. Receptor Binding Assay

Receptor binding specificity of representative ML/JS/1-1-965/2023(H4N6) was examined using the solid-phase direct binding assay, with *α*−2,3-siaylglycopolymer and *α*−2,6-Siaylglycopolymer, as described in a previous study [13]. Chicken antisera (ML/JS/1-1-965/2023(H4N6)) and a horseradish peroxidase-conjugated goat-anti-chicken antibody (Sigma–Aldrich, St. Louis, MO, USA) were used as the primary and secondary antibodies, respectively.

### 2.7. Viral Replication Kinetics In Vitro

MDCK cells, a commonly used canine kidney epithelial cell line, and 293T cells, a human renal embryonic epithelial cell line, were infected with the representative ML/JS/1-1-965/2023(H4N6) virus at a multiplicity of infection (MOI) of 0.01 for 1 hr, washed, overlaid with Opti-MEM containing 1 *μ*g/ml TPCK-treated trypsin and then cultured in a 5% CO_2_ incubator at 37°C. The supernatants were collected at hours 12, 24, 36, 48, 60, 72, 84, and 96 postinfection (p.i.), and viral titers were assessed in MDCK cells. Both MDCK and 293T cells were preserved in our laboratory.

### 2.8. Mouse Test

We used the representative ML/JS/1-1-965/2023(H4N6) for mouse experiments. After anesthesia with CO_2_, eight female 6-week-old BALB/c mice (Vital River Laboratories, Beijing, China) were inoculated intranasally with 10^6.0^ 50% egg infectious dose (EID_50_) of the virus in a 50 *μ*l volume, and the control group (five mice) was inoculated intranasally with 50 *μ*l PBS. Three mice in the inoculation group were euthanized on day 3 p.i., and the brains, nasal turbinates, spleens, kidneys, and lungs were collected for virus titration in 10-day-old chicken embryos. The virus titers in the organs were calculated by the Reed–Muench method [24]. Five mice, both in the inoculation group and control group, were monitored daily for survival and weight change until day 14 p.i..

## 3. Results

### 3.1. The H4N6 AIVs Isolated from Wild Birds in Jiangsu Province, China, 2023

During our routine surveillance of AIVs in March 2023, we collected 1,000 fresh fecal samples from wild bird habitats in Jiangsu Province, China, and subsequently isolated 20 AIVs from them, with an AIV isolation rate as 2% (*Supplementary 1*). Among the 20 viruses, five subtype combinations, including H1N1 (*n* = 4), H4N6 (*n* = 9), H5N3 (*n* = 1), H7N3 (*n* = 5), and H11N2 (*n* = 1), were identified by HI test and RT-PCR. The H4N6 subtype accounted for 45% (9 out of 20) of the AIVs isolated from wild birds. DNA barcoding analysis revealed that the hosts of our isolates were mallards belonging to the wild Anseriformes.

### 3.2. Phylogenetic Evolution Analysis of H4N6 AIVs from Wild Birds

**Figure 1:**
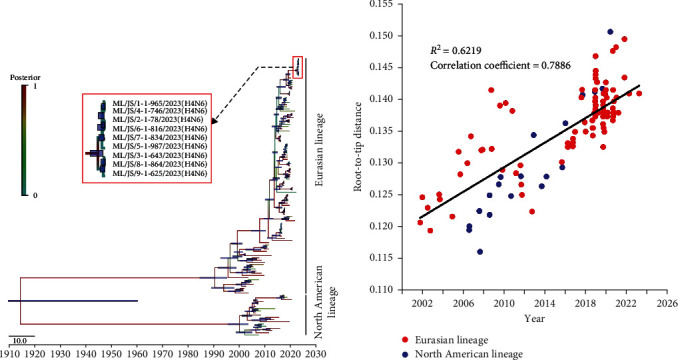
Bayesian analysis of the HA genes of H4N6 AIVs from wild birds. (a) MCC tree of the HA genes of H4N6 AIVs. The MCC tree is constructed by using the BEAST v1.10.4 software package. Node bars indicate 95% HPD of node height, and each branch is colored using posterior probability. The red frame represents our H4N6 isolates. (b) The temporal signal of the H4N6 AIVs root-to-tip regression analysis. The temporal signal of our dataset is detected by using TempEst v1.5.3 software.

To investigate the tMRCA (time to a most recent common ancestor) of H4N6 viruses in this study, we constructed the MCC trees (Figure 1(a), *Supplementary 2*) of eight segments of nine H4N6 viruses using BEAST v1.10.4. Before this, we investigated if the dataset used in this study contains sufficient temporal signals. As shown in Figure 1(b), a regression of root-to-tip genetic distance against sampling time (*R*^2^ = 0.6219, correlation coefficient = 0.7886) indicated that our HA dataset contained a significant temporal signal to further perform a time-calibrated phylogenetic analysis. The MCC tree (Figure 1(a)) revealed that the HA genes of H4N6 viruses were separated into two lineages, and our nine isolates fell into the Eurasian lineage. Bayesian phylogenetic analysis showed that the estimated mean tMRCA of eight segments (PB2, PB1, PA, HA, NP, NA, M, and NS) of nine H4N6 isolates were May 2022, Jul 2021, Apr 2021, Feb 2019, May 2021, Mar 2020, Jul 2020, and Nov 2021, respectively (Table 1).

According to the ML trees, the HA genes of nine H4N6 isolates clustered into the Eurasian lineage (*Supplementary 3*), which was consistent with the result of the MCC tree, with nucleotide identities of 100%. The NA genes of our nine isolates also belonged to the Eurasian lineage (*Supplementary 3*), with nucleotide identities of between 99.8% and 100%, and clustered with the NA gene of the H4N6 virus isolated from mallard in the Russian Federation. Phylogenetic analysis of the six internal genes of nine H4N6 viruses indicated that they fell into the Eurasian lineage (*Supplementary 4*) and showed high similarity, with PB2, PB1, PA, NP, M, and NS genes of our isolates sharing 99.6%–100%, 99.9%–100%, 99.7%–100%, 100%, 97.7%–99.7%, and 100% identity, respectively. Based on the phylogenetic analysis, the nine H4N6 isolates can be divided into one genotype, taking the nucleotide homology greater than 97% as the standard, and we select ML/JS/1-1-965/2023(H4N6) as the representative strain for further research.

### 3.3. The H4N6 Virus from Wild Birds Underwent Complex Gene Recombination

**Figure 2:**
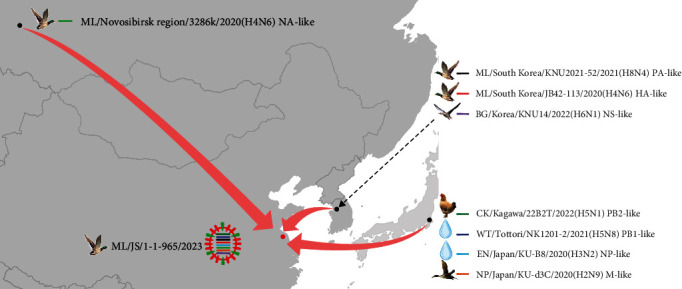
The schematic diagram of gene recombination of the representative ML/JS/1-1-965/2023(H4N6). The eight gene segments of the virus are represented by horizontal bars (from top to bottom, PB2, PB1, PA, HA, NP, NA, M, and NS).

We traced the origin of the representative ML/JS/1-1-965/2023(H4N6) by blasting the highest homology viruses in the NCBI Influenza Virus database and the GISAID EpiFlu database (Supplementary 5). As shown in Figure 2, its HA and NA genes were descended from H4N6 viruses isolated from mallard in South Korea (A/mallard/South Korea/JB42-113/2020(H4N6)-like) and in the Russian Federation (A/mallard/Novosibirsk region/3286 k/2020(H4N6)-like), respectively. For internal genes, PB2, PB1, NP, and M originated from Japan. It is worth noting that PB2 and PB1, among the polymerase complex, derived from clade 2.3.4.4b H5 HPAI viruses, with A/chicken/Kagawa/22B2T/2022(H5N1)-like and A/water/Tottori/NK1201-2/2021(H5N8)-like being their gene donors, respectively; its NP gene was introduced from an H3N2 virus isolated from environment (A/environment/Japan/KU-B8/2020(H3N2)-like), and its M gene was acquired from an H2N9 virus isolated from northern pintail (A/northern pintail/Japan/KU-d3C/2020(H2N9)-like). The remaining PA and NS genes were descended from LPAIVs in wild waterfowl in Korea, A/mallard(anas platyrhynchos)/South Korea/KNU2021-52/2021(H8N4)-like and A/bean goose(*Anser fabalis*)/Korea/KNU14/2022(H6N1)-like being their gene contributors, respectively.

Based on the above analysis, we found that the occurrence of these H4N6 viruses, represented by ML/JS/1-1-965/2023(H4N6) in this study was the result of complex recombination events, and their gene segments derived from different subtypes of AIVs from different countries. In particular, their PB2 and PB1 genes are descendants of the clade 2.3.4.4b H5 HPAI viruses from Japan.

### 3.4. The H4N6 AIVs from Wild Birds Possessed Molecular Signatures that Enhanced Pathogenicity in Mammals

Molecular analysis revealed that nine H4N6 isolates in this study possessed the same amino acid motif of PEKASR↓GLF in their HA cleavage site, indicating that they were LPAIVs [25]. It is worth noting that nine isolates contained the D3V and D622G mutations in the PB1 protein, N66S mutation in the PB1-F2 protein, N30D, I43M, and T215A mutations in the M1 protein, and P42S and I106M mutations in NS1 protein (Table 2), which are associated with enhanced AIVs pathogenicity in mice [26–33].

### 3.5. The H4N6 Virus from Wild Birds Acquired Dual Receptor Binding Ability

**Figure 3:**
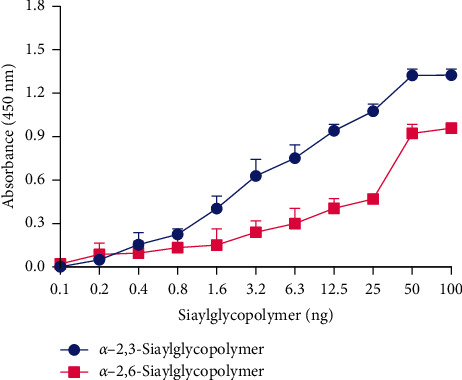
Receptor binding specificity of the representative ML/JS/1-1-965/2023(H4N6). The ability of the virus to bind to avian-type and human-type receptors was tested using two different glycans (*α*−2,3-siaylglycopolymer, blue; *α*−2,6-siaylglycopolymer, red). The data shown are the means of three repeats, and error bars represent the standard deviations.

We further examined the receptor binding preference of the representative ML/JS/1-1-965/2023(H4N6). As shown in Figure 3, the virus exhibited dual receptor-binding properties, and it could bind to both avian-type and human-type receptors.

### 3.6. The H4N6 Virus from Wild Birds Replicated Well in MDCK and 293T Cells

**Figure 4:**
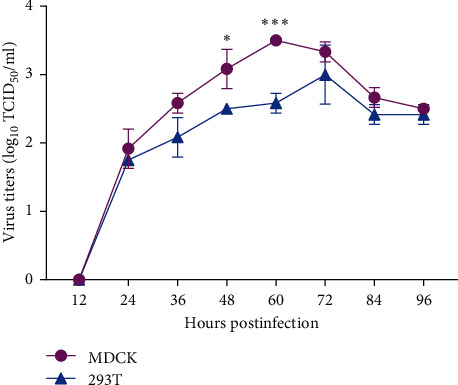
The growth curves of the representative ML/JS/1-1-965/2023(H4N6) in MDCK and 293T cells. MDCK and 293T monolayers were inoculated with the virus at an MOI of 0.01, and the supernatants were collected at the indicated time points and then titrated in MDCK cells. The statistical significance is calculated by Student's *t*-test using GraphPad Prism v8 ( ^*∗*^, *P*  < 0.05;  ^*∗∗∗*^, *P*  < 0.001). Three biological replicates are performed for each sample.

To explore the ability of the H4N6 virus to infect mammalian cells, MDCK, and 293T cells were infected with the representative ML/JS/1-1-965/2023(H4N6) at an MOI of 0.01 over a duration of 96 hr. As shown in Figure 4, the tested virus replicated well both in MDCK and 293T cells. It should be noted that this H4N6 virus replicated better in MDCK cells than in 293T cells, especially at hours 48 and 60 p.i. (about four and eight times, respectively) ( ^*∗*^, *P*  < 0.05;  ^*∗∗∗*^, *P*  < 0.001).

### 3.7. The H4N6 Virus from Wild Birds Could Infect BALB/c Mice without Prior Adaptation

**Figure 5:**
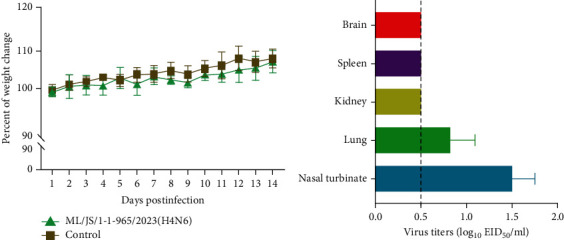
Pathogenicity and replication of the representative ML/JS/1-1-965/2023(H4N6) in mice. (a) Body weight changes of mice were monitored daily for 2 weeks. (b) Viral titers in the organs of mice on day 3 p.i. were detected. The dashed line indicates the lower limit of virus detection, and the error bars represent the standard deviation.

To investigate the potential threat of the H4N6 virus to mammals, we assessed the replication ability and virulence of the representative ML/JS/1-1-965/2023(H4N6) in BALB/c mice. As shown in Figure 5(a), the infected mice gradually gained weight during the observation period. In addition, on day 3 p.i., the virus could be detected in the lungs of two of three mice, with titers ranging from 0.5 to 0.98 log_10_ EID_50_, and in the turbinates of three mice, with titers ranging from 1.25 to 1.75 log_10_ EID_50_ (Figure 5(b)), while no virus was detected in the brain, spleen, or kidney tissues. These data indicate that the H4N6 virus isolated from wild birds can cross the species barrier to infect mice without prior adaptation.

## 4. Discussion

In March 2023, we isolated nine H4 subtype AIVs from 1,000 fresh fecal samples from wild bird habitats in Jiangsu Province, China, and their NA subtypes were N6. Previous study has shown that the H4N6 subtype is the dominant subtype combination of H4 viruses [42], which also happens in our study. Phylogenetic analysis showed that the eight gene segments of our H4N6 isolates were highly homologous, forming one genotype belonging to the Eurasian lineage.

Gene recombination is an important driving force for the evolution of AIVs [43]. Previous studies have shown that clade 2.3.4.4b HPAI H5N8 viruses are responsible for the new wave of outbreaks in poultry and wild birds at the beginning of 2020, causing substantial mortality among domestic poultry in Europe, Africa, and Asia [44]. During this period, the H5N8 viruses were reassorted with different AIVs and generated 2.3.4.4b HPAI H5N1 viruses, which further caused thousands of outbreaks in Europe, Africa, Asia, and North America [45]. Moreover, it is reported that H9N2 viruses can donate their internal segments to other subtypes of AIVs during reassortment, thereby enhancing the pathogenicity of the recombinant virus in mammals. Two novel reassortant H10N3 AIVs, with internal genes descended from genotype S (G57) H9N2 viruses, exhibited high pathogenicity in mice and could be effectively transmitted between guinea pigs [46]. Noteably, the PB2 and PB1 genes of the representative ML/JS/1-1-965/2023(H4N6) were descended from clade 2.3.4.4b H5N1 (A/chicken/Kagawa/22B2T/2022(H5N1)-like) and H5N8 (A/water/Tottori/NK1201-2/2021(H5N8)-like) HPAI viruses from Japan, respectively. These results demonstrate that the constant recombination between HPAIV and LPAIV clearly deserves close attention due to the innate recombination potential of AIVs and the long-distance migration of wild birds.

The switch in receptor binding specificity from avian-type to human-type sialic acid receptors is an important mutation of AIVs, which is considered to be one of the major determinants of its efficient replication in human hosts [47]. In this study, the tested H4N6 virus isolated from wild birds exhibited dual receptor binding preferences, with a certain ability to bind to human-type receptors, which is similar to the receptor binding properties of H4 viruses isolated from domestic ducks [13]. However, whether the H4N6 viruses isolated from chickens also have dual receptor binding properties deserves further exploration. These data highlight the potential threat of H4N6 viruses to mammals.

Influenza A virus is prone to adaptive mutations during their evolution process to replicate efficiently in mammals [48]. In this study, nine H4N6 isolates contain the D3V and D622G mutations in the PB1 protein, N66S mutation in the PB1-F2 protein, N30D, I43M, and T215A mutations in the M1 protein, and P42S and I106 M mutations in NS1 protein, which may contribute to increased virulence of the viruses in mice. To investigate the ability of the H4N6 virus isolated from wild birds to infect mammals, we conducted both in vitro and in vivo experiments. The results showed that the tested virus could replicate well in mammalian cells, including MDCK and 293T cells. Also, the tested virus acquired the ability to replicate in the mammalian model, the BALB/c mice, without prior adaptation. These data suggest that the H4N6 virus from wild birds acquired several mammalian adaptive mutations and could efficiently replicate in mammalian cells and mice, which posed a potential threat to public health security. Thus, we should continue to pay attention to whether the H4N6 virus will cross the interspecies barrier to infect humans.

## 5. Conclusions

In summary, the H4N6 viruses newly isolated from wild birds were recombinant from different subtypes of AIVs among Asian countries, especially their PB2 and PB1 segments derived from H5 HPAI viruses, and the genome possessed molecular signatures that enhanced the pathogenicity in mammals. Our data revealed that the representative H4N6 virus exhibited dual receptor binding properties and could be replicated both in mammalian cells, including MDCK and 293T cells, and in BALB/c mice. Therefore, we should focus on the public health concern represented by H4N6 viruses, and we propose that active monitoring of H4N6 viruses from wild birds should be strengthened.

## Figures and Tables

**Table 1:** The tMRCA of eight segments of H4N6 AIVs from wild birds.

Segment	Correlation coefficient	tMRCA	95% HPD^a^ interval	Posterior probability
PB2	0.9391	May. 2022	Mar. 2022 to Nov. 2023	0.9997
PB1	0.8940	Jul. 2021	Jul. 2021 to Jun. 2022	0.9517
PA	0.8780	Apr. 2021	Jan. 2021 to Sep. 2022	0.9938
HA	0.7886	Feb. 2019	Feb. 2019 to Sep. 2020	0.9386
NP	0.8067	May. 2021	May. 2021 to Jun. 2022	0.8734
NA	0.9411	Mar. 2020	Feb. 2020 to Sep. 2021	0.8866
M	0.5564	Jul. 2020	Jul. 2020 to Jul. 2021	0.9935
NS	0.9316	Nov. 2021	Nov. 2021 to Apr. 2023	0.7626

^a^HPD, highest posterior density.

**Table 2:** Molecular markers of H4N6 AIVs from wild birds in the study.

Protein	Amino acid position/motif	Phenotypic consequences	References	Isolates in the study
PB2	E158K	Increased virulence in mice and increased polymerase activity	[34]	—^b^
	E627K	Increased virulence in mice and increased polymerase activity	[34]	—
	D701N	Increased virulence in mice and increased polymerase activity	[35]	—

PB1	D3V	Increased virulence in mice and increased polymerase activity	[26]	V
	D622G	Increased virulence in mice and increased polymerase activity	[27]	G

PB1-F2	N66S	Increased virulence in mice	[28, 29]	S

HA (H3 numbering)^a^	E190G	Increased virus binding to human-type receptor	[36]	—
	G225D	Increased virus binding to human-type receptor	[37]	—
	Q226L	Increased virus binding to human-type receptor	[18]	—
	G228A/S	Increased virus binding to human-type receptor	[18]	—
	T187P + M227L	Increased virulence in mice	[38]	—

NA (N2 numbering)^a^	Stalk deletion	Increased virulence in mice	[39]	—
	E119A/D/G	Reduced susceptibility to zanamivir	[40]	—
	R292 K	Reduced susceptibility to zanamivir	[40]	—

M1	N30D	Increased virulence in mice	[30]	D
	I43M	Increased virulence in mice	[31]	M
	T215A	Increased virulence in mice	[30]	A

NS1	80–84 Deletion	Increased virulence in mice	[41]	—
	P42S	Increased virulence in mice	[32]	S
	I106M	Increased virulence in mice	[33]	M

^a^The mutations/motifs are numbered according to alignments with A/Aichi/2/1968(H3N2). ^b^The “−” indicates the mutation was not detected in the H4N6 viruses.

## Data Availability

The genome sequences of the nine H4N6 viruses were deposited in the GISAID EpiFlu database (accession numbers: EPI2792671–EPI2792742).

## Supplementary Materials

Supplementary 1 Avian influenza viruses isolated from wild birds in March 2023 in China.

Supplementary 2 MCC trees of the remaining seven segments except the HA gene of H4N6 AIVs from wild birds.

Supplementary 3 ML phylogenetic trees of the external genes of H4N6 AIVs from wild birds.

Supplementary 4 ML phylogenetic trees of the internal genes of H4N6 AIVs from wild birds.

Supplementary 5 The highest nucleotide homology of the whole genomes of representative H4N6 virus.
