# Supplementary material for: Genetics and Pathogenicity of Influenza A (H4N6) Virus Isolated from Wild Birds in Jiangsu Province, China, 2023
Source: Transbound Emerg Dis. 2024 Feb 14;2024:7421277. doi: 10.1155/2024/7421277 (PMC12017178; doi:10.1155/2024/7421277)
Supplement: Supplementary 1 — Avian influenza viruses isolated from wild birds in March 2023 in China. [file 7421277.f1.docx]

**Table S1**. Avian influenza viruses isolated from wild birds in March 2023 in China.

| **Sample** | | **AIV isolate** | | | **H4N6 AIV** | |
| --- | --- | --- | --- | --- | --- | --- |
| **No.** | **Type** | **No.** | **Positive rate (%)** | **Subtype (No.)** | **Name** | **Abbreviation** |
| 1000 | Feces | 20 | 2 | H4N6 (9) | A/mallard/JS/1-1-965/2023 | ML/JS/1-1-965/2023 |
|  |  |  |  |  | A/mallard/JS/2-1-78/2023 | ML/JS/2-1-78/2023 |
|  |  |  |  |  | A/mallard/JS/3-1-643/2023 | ML/JS/3-1-643/2023 |
|  |  |  |  |  | A/mallard/JS/4-1-746/2023 | ML/JS/4-1-746/2023 |
|  |  |  |  |  | A/mallard/JS/5-1-987/2023 | ML/JS/5-1-987/2023 |
|  |  |  |  |  | A/mallard/JS/6-1-816/2023 | ML/JS/6-1-816/2023 |
|  |  |  |  |  | A/mallard/JS/7-1-834/2023 | ML/JS/7-1-834/2023 |
|  |  |  |  |  | A/mallard/JS/8-1-864/2023 | ML/JS/8-1-864/2023 |
|  |  |  |  |  | A/mallard/JS/9-1-625/2023 | ML/JS/9-1-625/2023 |
|  |  |  |  | Multiple^a^ (11) |  |  |

a: The 11 AIVs are 4 H1N1, 1 H5N3, 5 H7N3 and 1 H11N2 strains.
