# Supplementary figures and images for: Genetics and Pathogenicity of Influenza A (H4N6) Virus Isolated from Wild Birds in Jiangsu Province, China, 2023
Source: Transbound Emerg Dis. 2024 Feb 14;2024:7421277. doi: 10.1155/2024/7421277 (PMC12017178; doi:10.1155/2024/7421277)

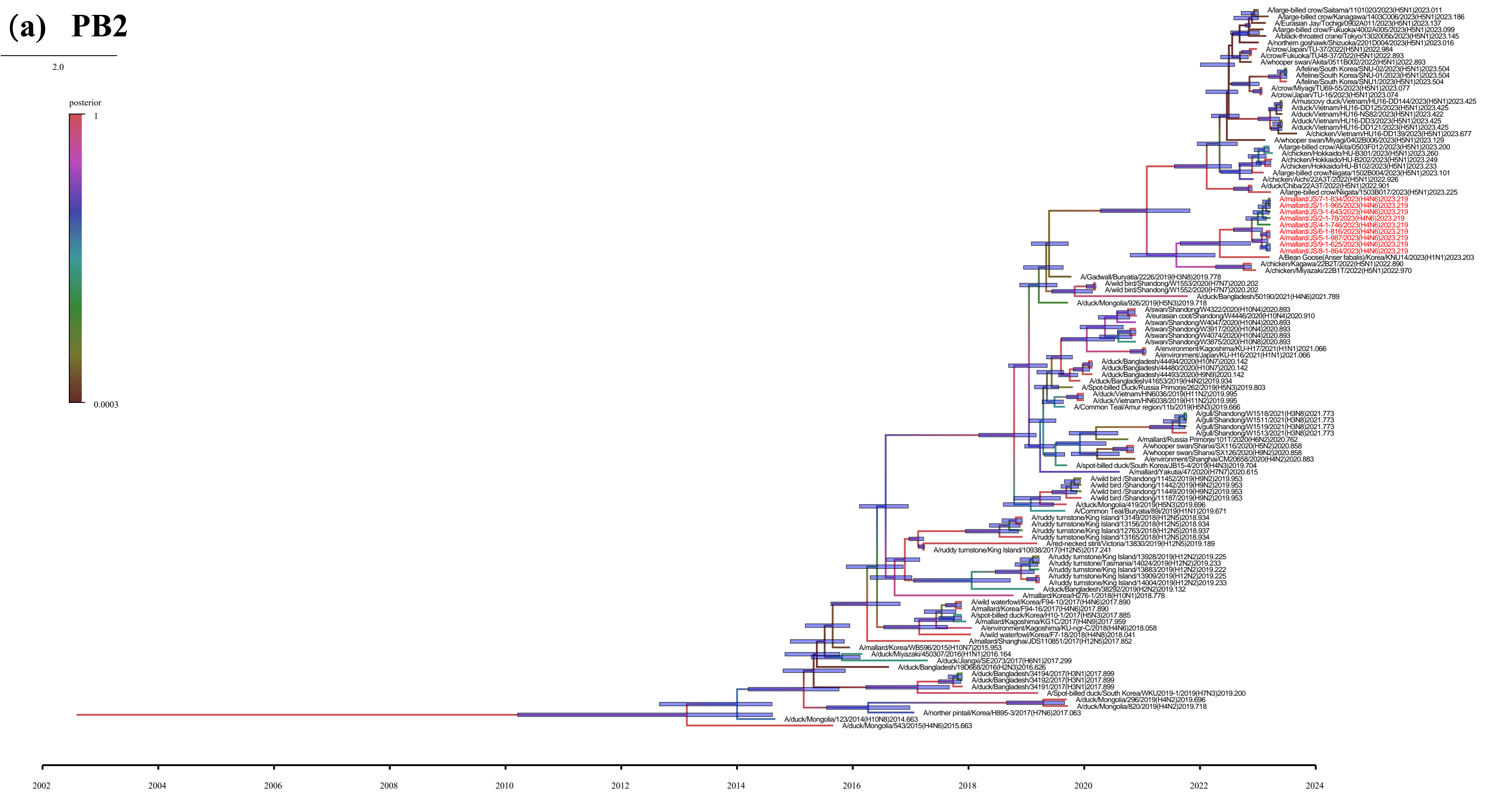

(b) PB1

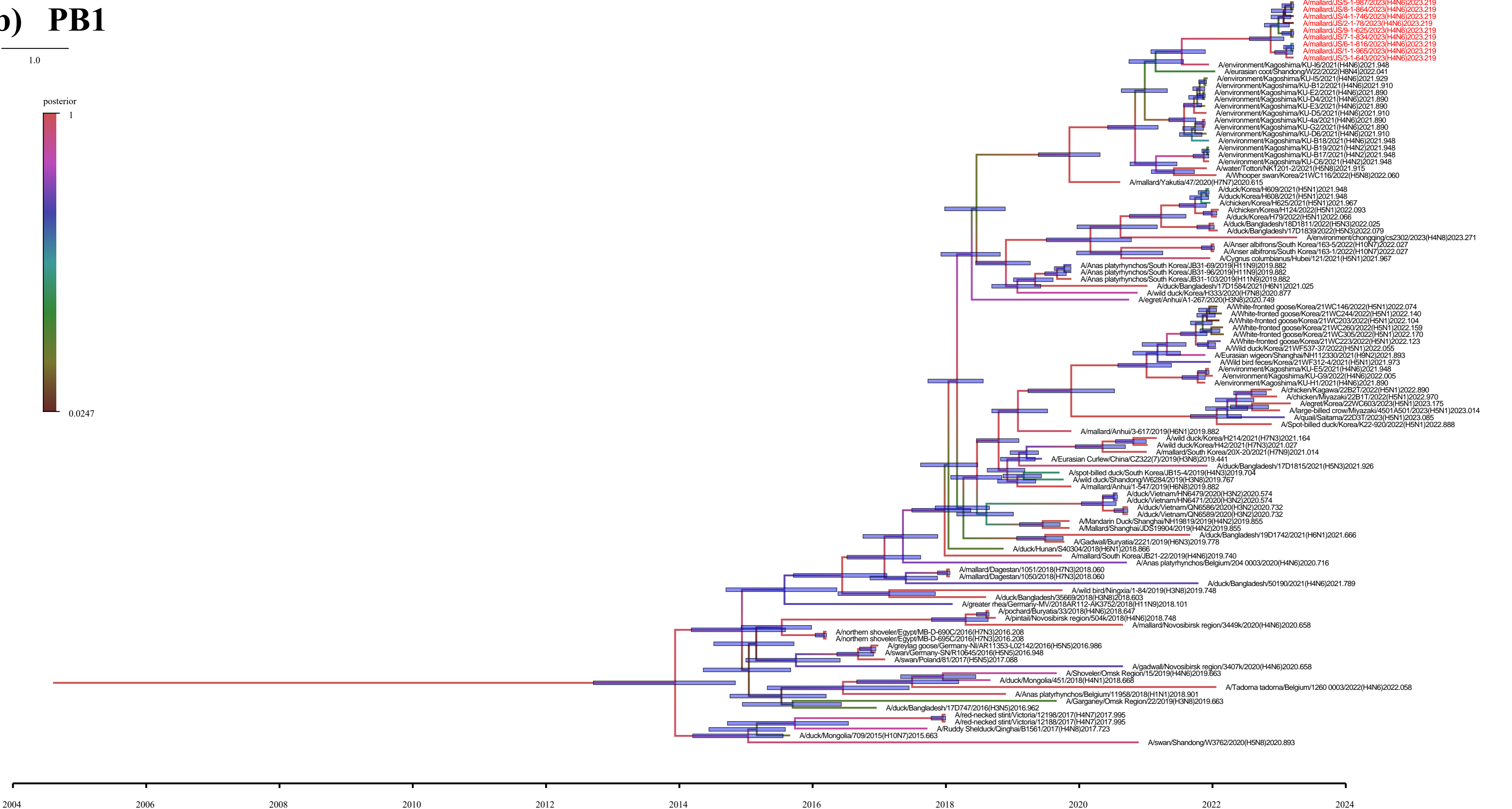

(c) PA

2.0

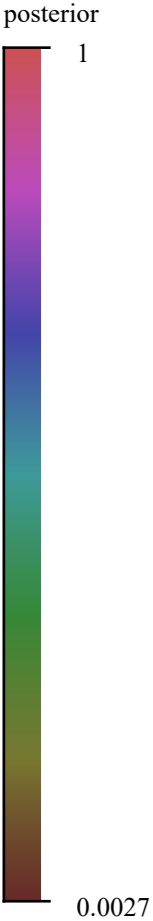

1988 1990 1992 1994 1996 1998 2000 2002 2004 2006 2008 2010 2012 2014 2016 2018 2020 2022 2024

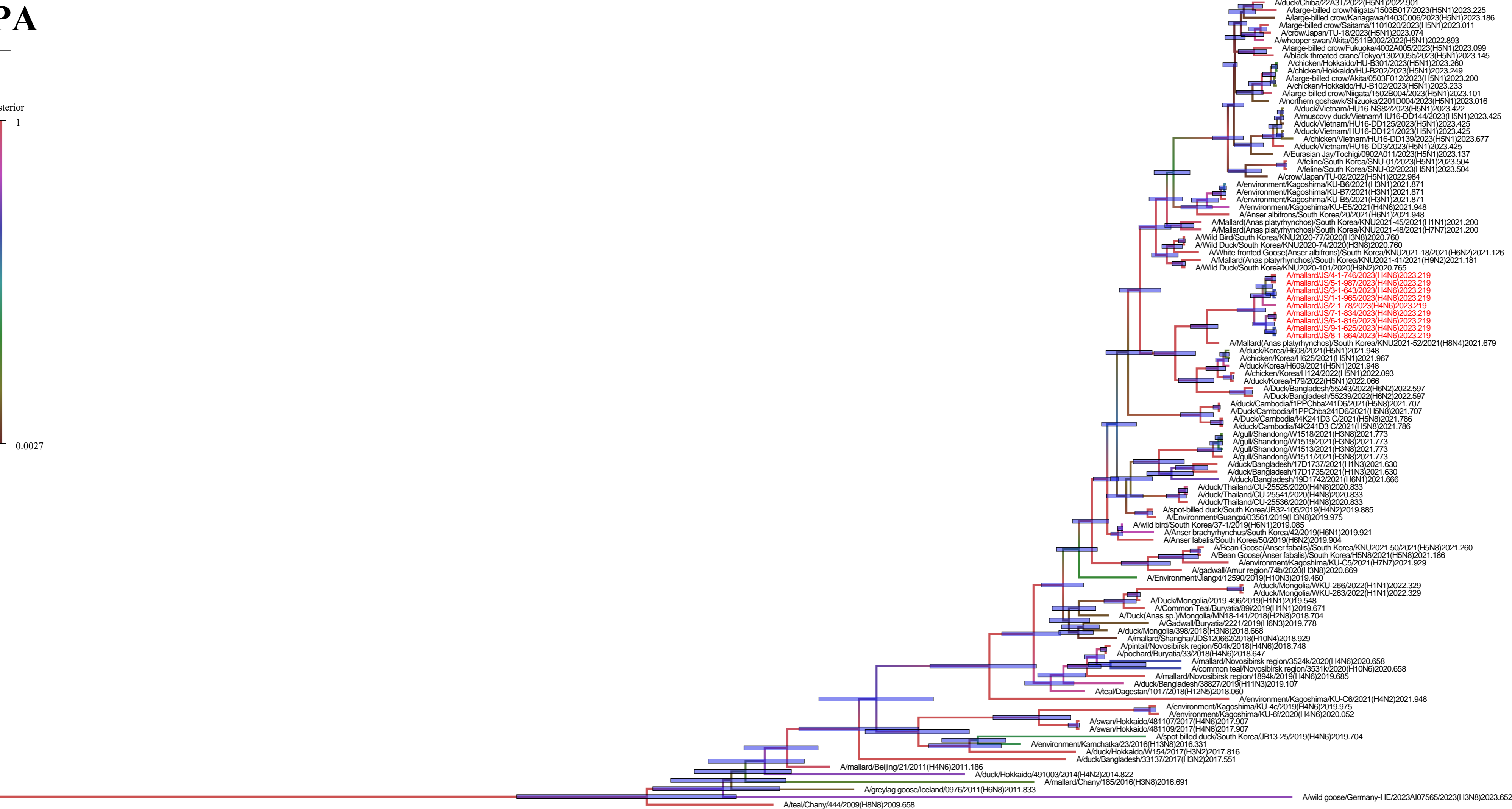

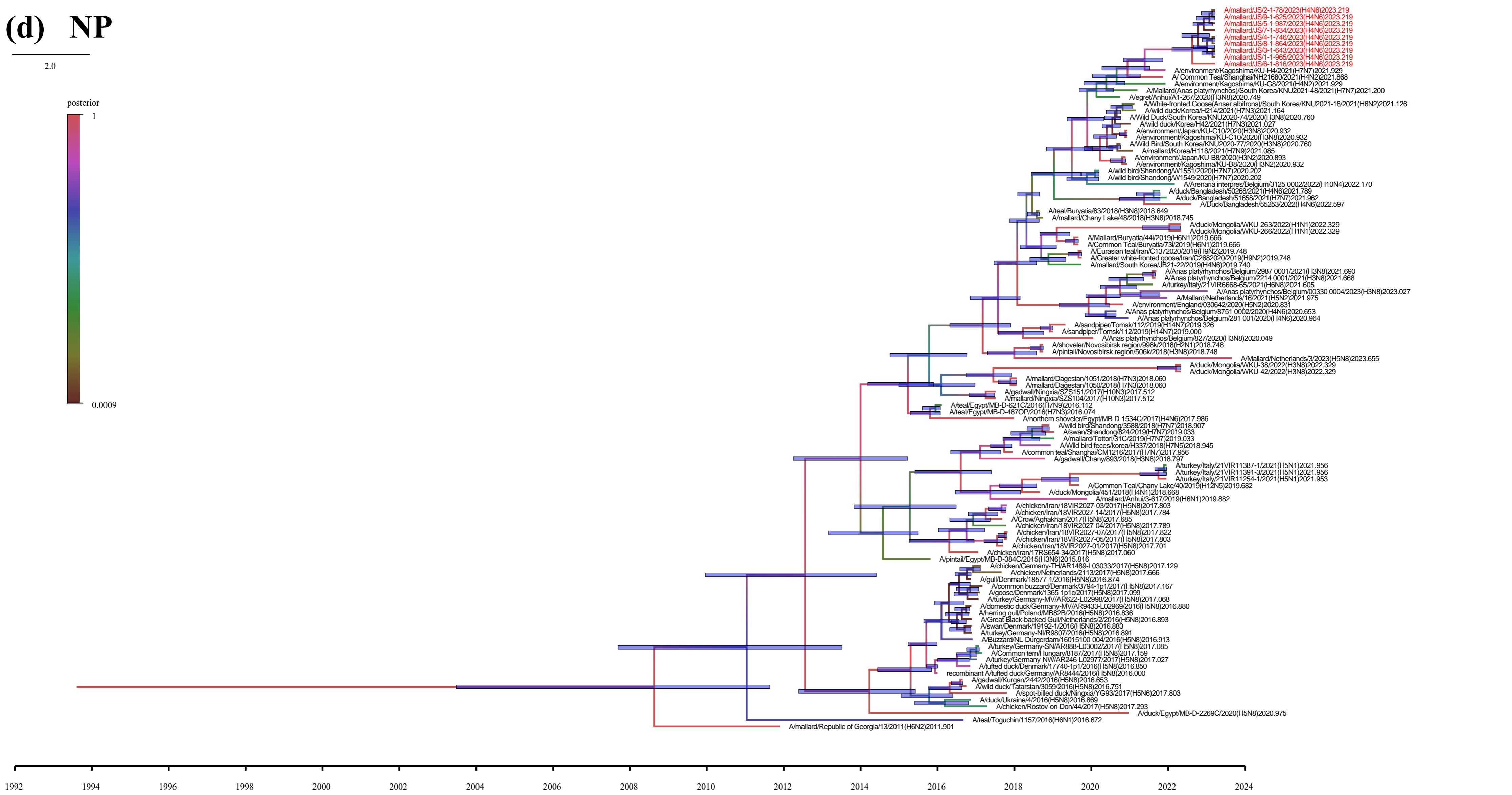

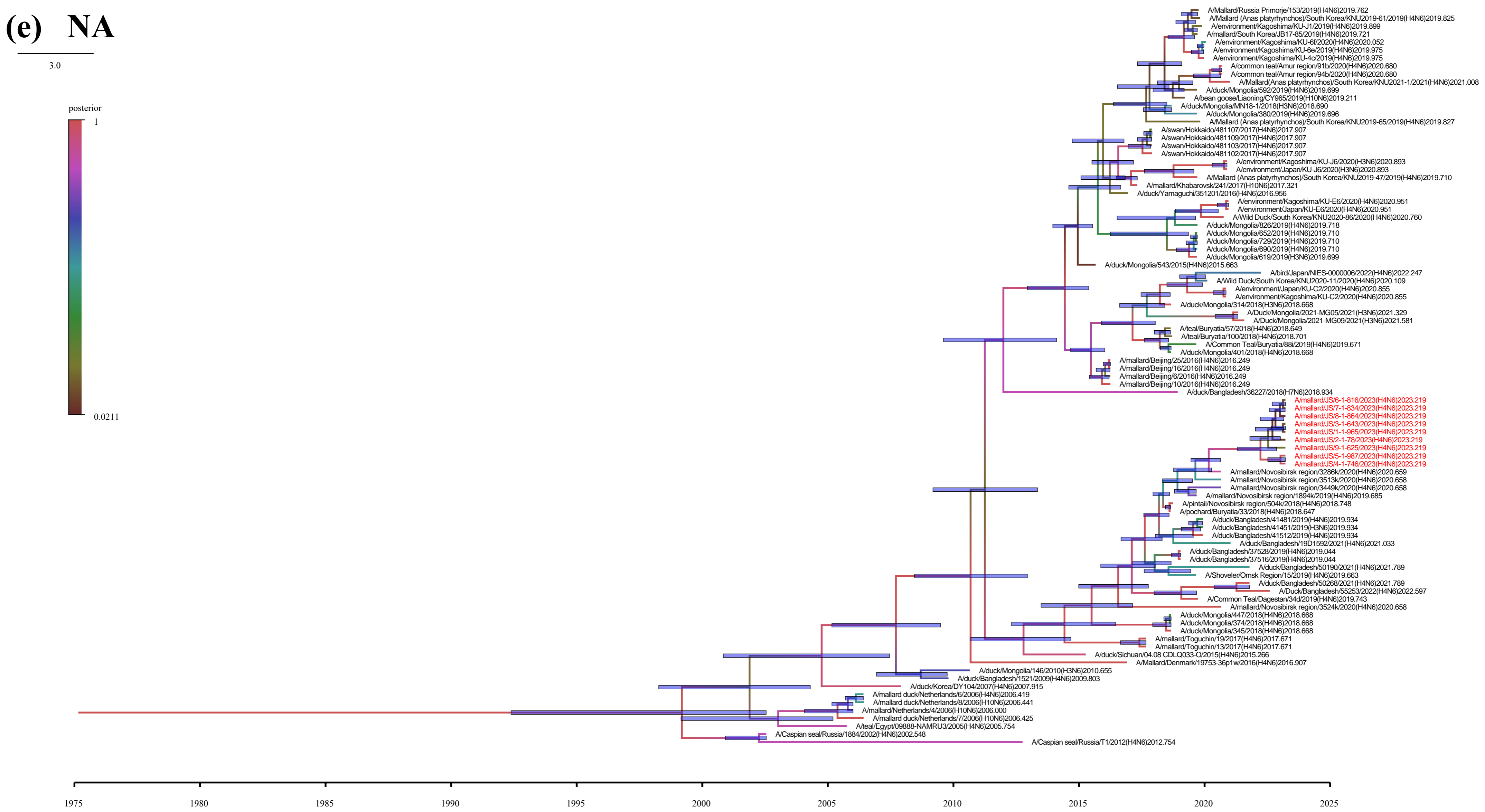

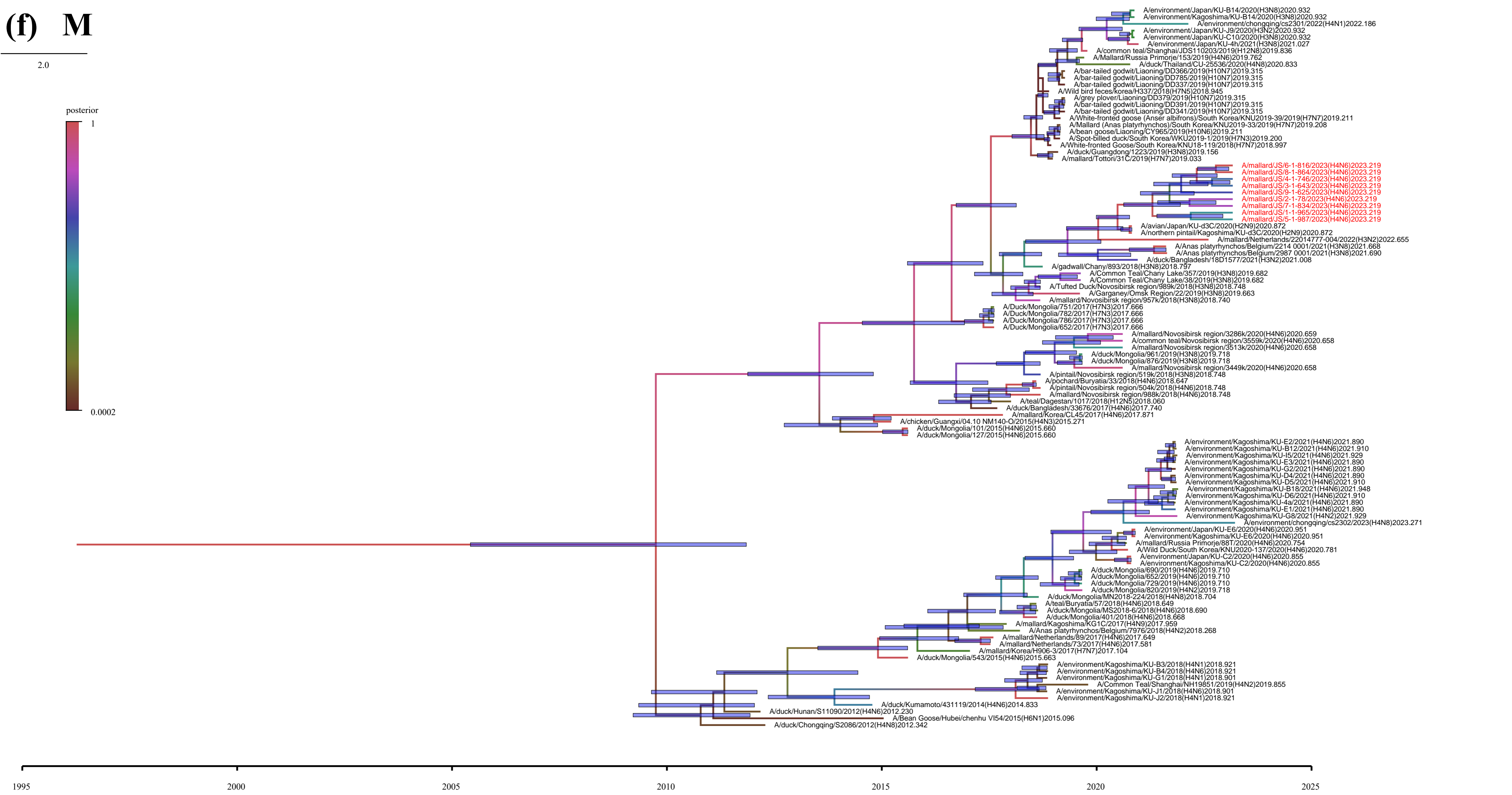

2.0

2.0

posterior

1

0.0001

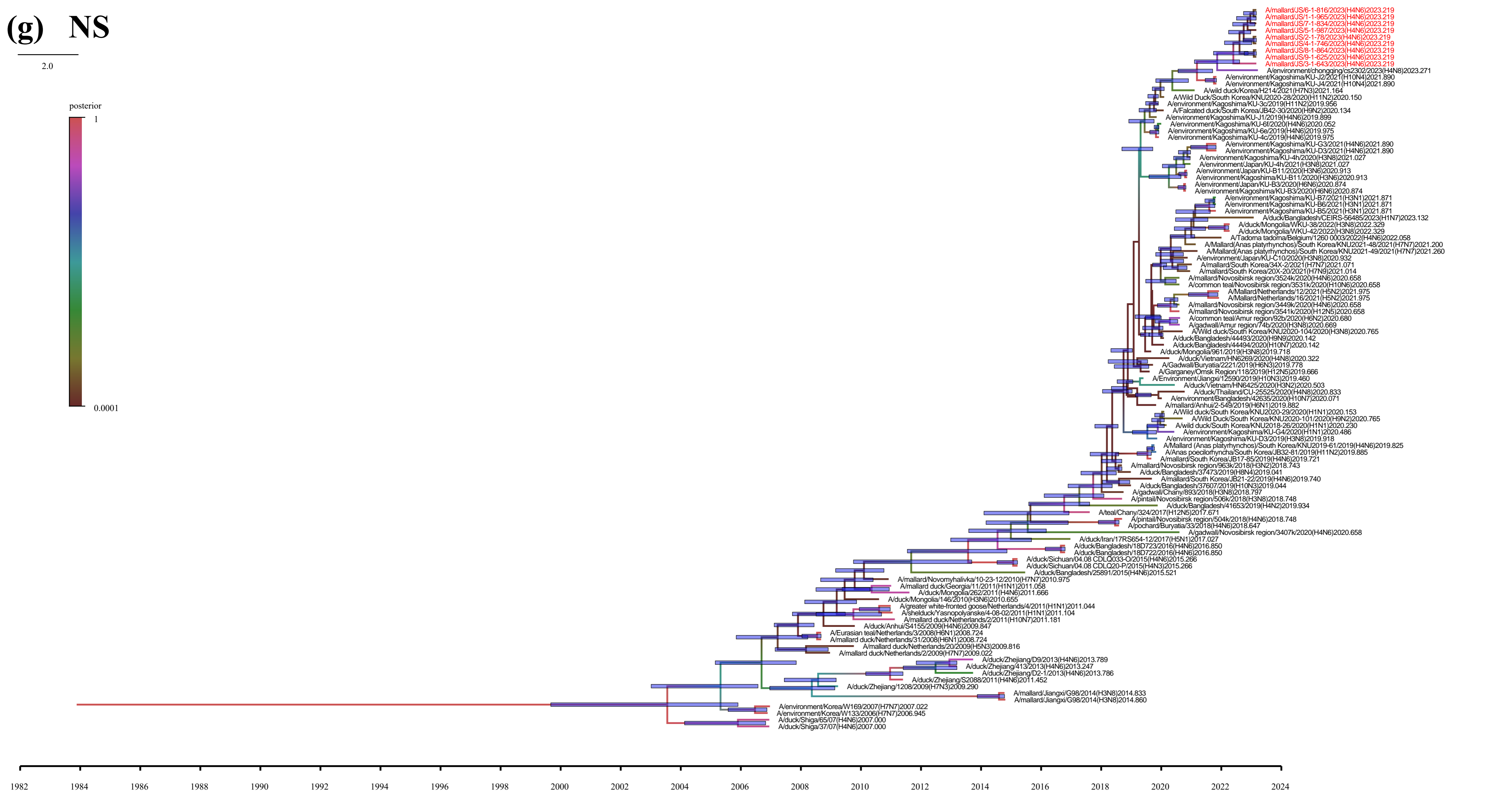

Supplement: Supplementary 2 — MCC trees of the remaining seven segments except the HA gene of H4N6 AIVs from wild birds. [file 7421277.f2.pdf]

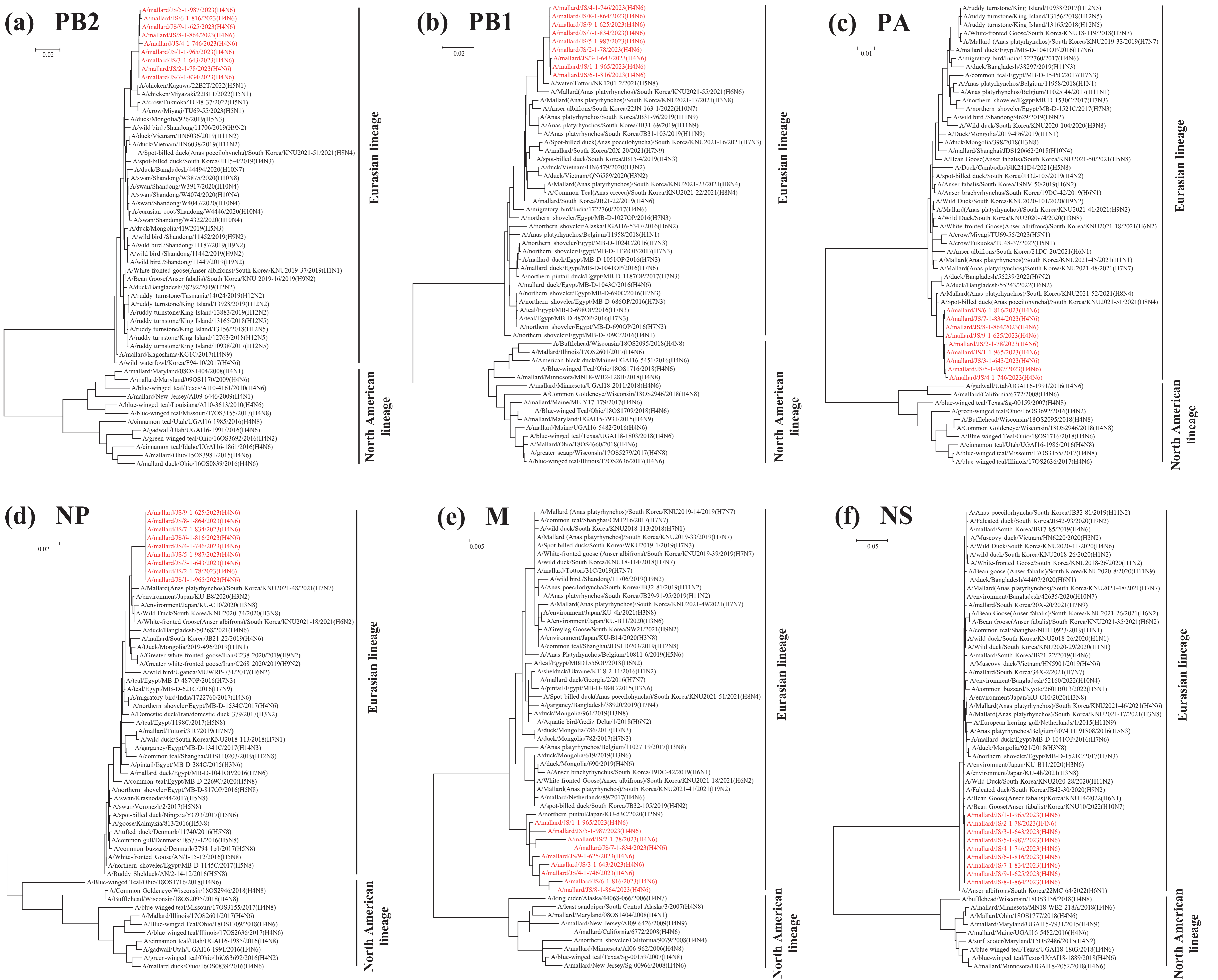

Supplement: Supplementary 4 — ML phylogenetic trees of the internal genes of H4N6 AIVs from wild birds. [file 7421277.f4.pdf]
