# Supplementary material for: Genetics and Pathogenicity of Influenza A (H4N6) Virus Isolated from Wild Birds in Jiangsu Province, China, 2023
Source: Transbound Emerg Dis. 2024 Feb 14;2024:7421277. doi: 10.1155/2024/7421277 (PMC12017178; doi:10.1155/2024/7421277)
Supplement: Supplementary 5 — The highest nucleotide homology of the whole genomes of representative H4N6 virus. [file 7421277.f5.docx]

**Table S2**. The highest nucleotide homology of the whole genomes of representative H4N6 virus.

| **Segment** | **Highest homologous strain** |  |  |  |
| --- | --- | --- | --- | --- |
|  | **Name** | **Abbreviation** | **Homology** | **Accession** |
| PB2 | A/chicken/Kagawa/22B2T/2022(H5N1) | CK/Kagawa/22B2T/2022(H5N1) | 98.90% | EPI2747727 |
| PB1 | A/water/Tottori/NK1201-2/2021(H5N8) | WT/Tottori/NK1201-2/2021(H5N8) | 99.25% | LC699142.1 |
| PA | A/mallard(anas platyrhynchos)/South Korea/KNU2021-52/2021(H8N4) | ML/South Korea/KNU2021-52/2021(H8N4) | 99.44% | ON505926.1 |
| HA | A/mallard/South Korea/JB42-113/2020(H4N6) | ML/South Korea/JB42-113/2020(H4N6) | 99.23% | MW494156.1 |
| NP | A/environment/Japan/KU-B8/2020(H3N2) | EN/Japan/KU-B8/2020(H3N2) | 99.06% | OR048414.1 |
| NA | A/mallard/Novosibirsk region/3286k/2020(H4N6) | ML/Novosibirsk region/3286k/2020(H4N6) | 98.20% | EPI1849884 |
| M | A/northern pintail/Japan/KU-d3C/2020(H2N9) | NP/Japan/KU-d3C/2020(H2N9) | 99.49% | OR048716.1 |
| NS | A/bean goose(anser fabalis)/Korea/KNU14/2022(H6N1) | BG/Korea/KNU14/2022(H6N1) | 99.76% | OR674067.1 |
