# Supplementary material for: Genetics and Pathogenicity of Influenza A (H4N6) Virus Isolated from Wild Birds in Jiangsu Province, China, 2023
Source: Transbound Emerg Dis. 2024 Feb 14;2024:7421277. doi: 10.1155/2024/7421277 (PMC12017178; doi:10.1155/2024/7421277)
Supplement: Supplementary 3 — ML phylogenetic trees of the external genes of H4N6 AIVs from wild birds. [file 7421277.f3.pdf]

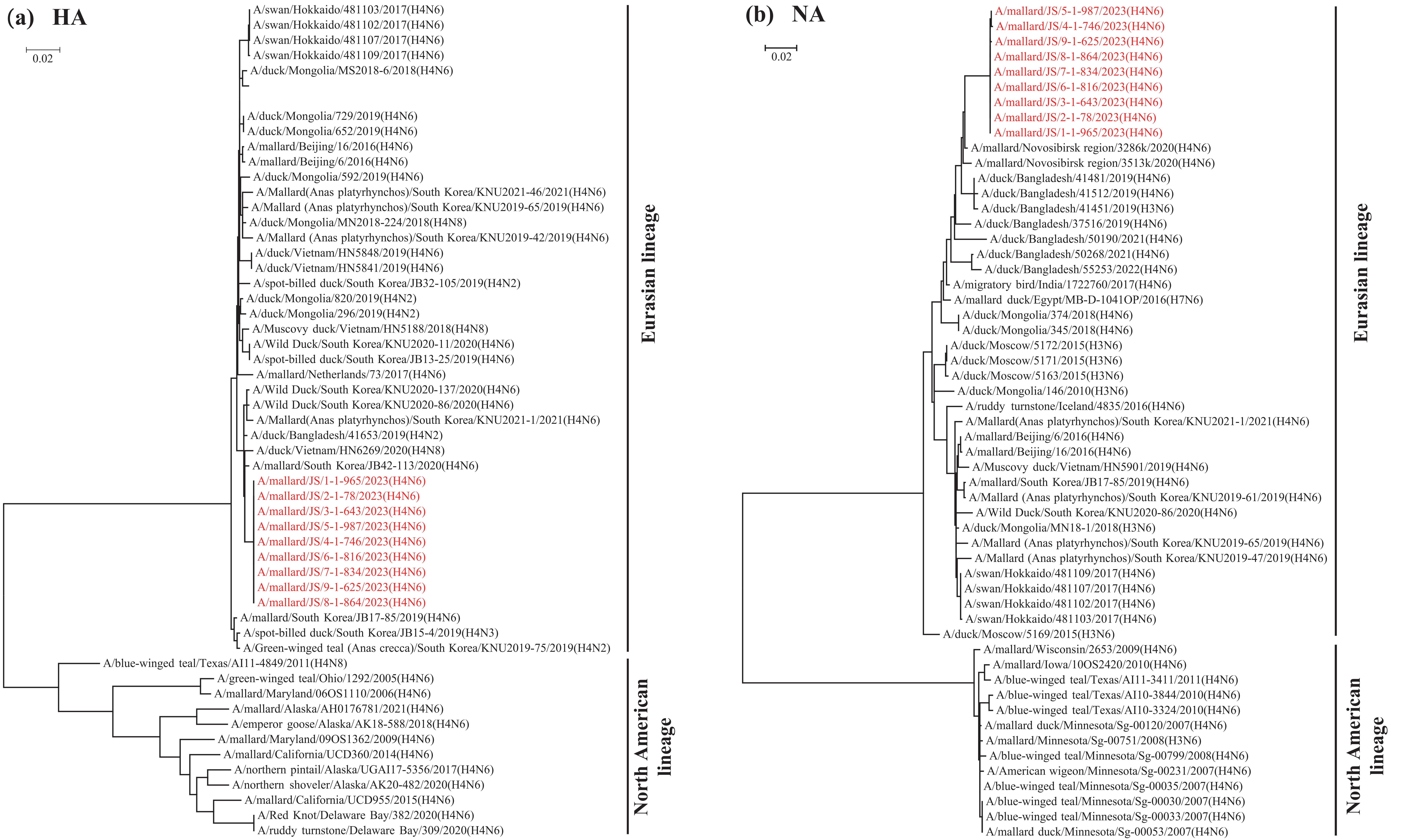

(a) HA
0.02
A/swan/Hokkaido/481103/2017(H4N6)
A/swan/Hokkaido/481102/2017(H4N6)
A/swan/Hokkaido/481107/2017(H4N6)
A/swan/Hokkaido/481109/2017(H4N6)
A/duck/Mongolia/MS2018-6/2018(H4N6)
A/duck/Mongolia/729/2019(H4N6)
A/duck/Mongolia/652/2019(H4N6)
A/mallard/Beijing/16/2016(H4N6)
A/mallard/Beijing/6/2016(H4N6)
A/duck/Mongolia/592/2019(H4N6)
A/Mallard(Anas platyrhynchos)/South Korea/KNU2021-46/2021(H4N6)
A/Mallard (Anas platyrhynchos)/South Korea/KNU2019-65/2019(H4N6)
A/duck/Mongolia/MN2018-224/2018(H4N8)
A/Mallard (Anas platyrhynchos)/South Korea/KNU2019-42/2019(H4N6)
A/duck/Vietnam/HN5848/2019(H4N6)
A/duck/Vietnam/HN5841/2019(H4N6)
A/spot-billed duck/South Korea/JB32-105/2019(H4N2)
A/duck/Mongolia/820/2019(H4N2)
A/duck/Mongolia/296/2019(H4N2)
A/Muscovy duck/Vietnam/HN5188/2018(H4N8)
A/Wild Duck/South Korea/KNU2020-11/2020(H4N6)
A/spot-billed duck/South Korea/JB13-25/2019(H4N6)
A/mallard/Netherlands/73/2017(H4N6)
A/Wild Duck/South Korea/KNU2020-137/2020(H4N6)
A/Wild Duck/South Korea/KNU2020-86/2020(H4N6)
A/Mallard(Anas platyrhynchos)/South Korea/KNU2021-1/2021(H4N6)
A/duck/Bangladesh/41653/2019(H4N2)
A/duck/Vietnam/HN6269/2020(H4N8)
A/mallard/South Korea/JB42-113/2020(H4N6)
A/mallard/JS/1-1-965/2023(H4N6)
A/mallard/JS/2-1-78/2023(H4N6)
A/mallard/JS/3-1-643/2023(H4N6)
A/mallard/JS/5-1-987/2023(H4N6)
A/mallard/JS/4-1-746/2023(H4N6)
A/mallard/JS/6-1-816/2023(H4N6)
A/mallard/JS/7-1-834/2023(H4N6)
A/mallard/JS/9-1-625/2023(H4N6)
A/mallard/JS/8-1-864/2023(H4N6)
A/mallard/South Korea/JB17-85/2019(H4N6)
A/spot-billed duck/South Korea/JB15-4/2019(H4N3)
A/Green-winged teal (Anas crecca)/South Korea/KNU2019-75/2019(H4N2)
A/blue-winged teal/Texas/AI11-4849/2011(H4N8)
A/green-winged teal/Ohio/1292/2005(H4N6)
A/mallard/Maryland/06OS1110/2006(H4N6)
A/mallard/Alaska/AH0176781/2021(H4N6)
A/emperor goose/Alaska/AK18-588/2018(H4N6)
A/mallard/Maryland/09OS1362/2009(H4N6)
A/mallard/California/UCD360/2014(H4N6)
A/northern pintail/Alaska/UGAI17-5356/2017(H4N6)
A/northern shoveler/Alaska/AK20-482/2020(H4N6)
A/mallard/California/UCD955/2015(H4N6)
A/Red Knot/Delaware Bay/382/2020(H4N6)
A/ruddy turnstone/Delaware Bay/309/2020(H4N6)
Eurasian lineage
North American lineage
(b) NA
0.02
A/mallard/JS/5-1-987/2023(H4N6)
A/mallard/JS/4-1-746/2023(H4N6)
A/mallard/JS/9-1-625/2023(H4N6)
A/mallard/JS/8-1-864/2023(H4N6)
A/mallard/JS/7-1-834/2023(H4N6)
A/mallard/JS/6-1-816/2023(H4N6)
A/mallard/JS/3-1-643/2023(H4N6)
A/mallard/JS/2-1-78/2023(H4N6)
A/mallard/JS/1-1-965/2023(H4N6)
A/mallard/Novosibirsk region/3286k/2020(H4N6)
A/mallard/Novosibirsk region/3513k/2020(H4N6)
A/duck/Bangladesh/41481/2019(H4N6)
A/duck/Bangladesh/41512/2019(H4N6)
A/duck/Bangladesh/41451/2019(H3N6)
A/duck/Bangladesh/37516/2019(H4N6)
A/duck/Bangladesh/50190/2021(H4N6)
A/duck/Bangladesh/50268/2021(H4N6)
A/duck/Bangladesh/55253/2022(H4N6)
A/migratory bird/India/1722760/2017(H4N6)
A/mallard duck/Egypt/MB-D-1041OP/2016(H7N6)
A/duck/Mongolia/374/2018(H4N6)
A/duck/Mongolia/345/2018(H4N6)
A/duck/Moscow/5172/2015(H3N6)
A/duck/Moscow/5171/2015(H3N6)
A/duck/Moscow/5163/2015(H3N6)
A/duck/Mongolia/146/2010(H3N6)
A/ruddy turnstone/Iceland/4835/2016(H4N6)
A/Mallard(Anas platyrhynchos)/South Korea/KNU2021-1/2021(H4N6)
A/mallard/Beijing/6/2016(H4N6)
A/mallard/Beijing/16/2016(H4N6)
A/Muscovy duck/Vietnam/HN5901/2019(H4N6)
A/mallard/South Korea/JB17-85/2019(H4N6)
A/Mallard (Anas platyrhynchos)/South Korea/KNU2019-61/2019(H4N6)
A/Wild Duck/South Korea/KNU2020-86/2020(H4N6)
A/duck/Mongolia/MN18-1/2018(H3N6)
A/Mallard (Anas platyrhynchos)/South Korea/KNU2019-65/2019(H4N6)
A/Mallard (Anas platyrhynchos)/South Korea/KNU2019-47/2019(H4N6)
A/swan/Hokkaido/481109/2017(H4N6)
A/swan/Hokkaido/481107/2017(H4N6)
A/swan/Hokkaido/481102/2017(H4N6)
A/swan/Hokkaido/481103/2017(H4N6)
A/duck/Moscow/5169/2015(H3N6)
A/mallard/Wisconsin/2653/2009(H4N6)
A/mallard/Iowa/10OS2420/2010(H4N6)
A/blue-winged teal/Texas/AI11-3411/2011(H4N6)
A/blue-winged teal/Texas/AI10-3844/2010(H4N6)
A/blue-winged teal/Texas/AI10-3324/2010(H4N6)
A/mallard duck/Minnesota/Sg-00120/2007(H4N6)
A/mallard/Minnesota/Sg-00751/2008(H3N6)
A/blue-winged teal/Minnesota/Sg-00799/2008(H4N6)
A/American wigeon/Minnesota/Sg-00231/2007(H4N6)
A/blue-winged teal/Minnesota/Sg-00035/2007(H4N6)
A/blue-winged teal/Minnesota/Sg-00030/2007(H4N6)
A/blue-winged teal/Minnesota/Sg-00033/2007(H4N6)
A/mallard duck/Minnesota/Sg-00053/2007(H4N6)
Eurasian lineage
North American lineage
